# Proanthocyanidin oxidation of *Arabidopsis* seeds is altered in mutant of the high-affinity nitrate transporter NRT2.7

**DOI:** 10.1093/jxb/ert481

**Published:** 2014-02-13

**Authors:** Laure C. David, Julie Dechorgnat, Patrick Berquin, Jean Marc Routaboul, Isabelle Debeaujon, Françoise Daniel-Vedele, Sylvie Ferrario-Méry

**Affiliations:** ^1^Institut Jean-Pierre Bourgin (IJPB), UMR 1318 INRA-AgroParisTech, Centre de Versailles-Grignon, Route de St-Cyr (RD10), F-78026 Versailles cedex, France; ^2^University of Adelaide, School of Agriculture Food and Wine, PRC, 2B Hartley Grove, Urrbrae, SA 5064, Australia; ^3^Genomic and Biotechnology of Fruit, UMR 990 INRA/INP-ENSAT, 24, Chemin de Borderouge-Auzeville CS 52627, F-31326 Castanet-Tolosan cedex, France

**Keywords:** Flavonoids, laccase, nitrate, NRT2.7, proanthocyanidins, seeds, transporter, TT1.0.

## Abstract

NRT2.7 is a seed-specific high-affinity nitrate transporter controlling nitrate content in *Arabidopsis* mature seeds. The objective of this work was to analyse further the consequences of the *nrt2.7* mutation for the seed metabolism. This work describes a new phenotype for the *nrt2.7-2* mutant allele in the Wassilewskija accession, which exhibited a distinctive pale-brown seed coat that is usually associated with a defect in flavonoid oxidation. Indeed, this phenotype resembled those of *tt10* mutant seeds defective in the laccase-like enzyme TT10/LAC15, which is involved in the oxidative polymerization of flavonoids such as the proantocyanidins (PAs) (i.e. epicatechin monomers and PA oligomers) and flavonol glycosides. *nrt2.7-2* and *tt10-2* mutant seeds displayed the same higher accumulation of PAs, but were partially distinct, since flavonol glycoside accumulation was not affected in the *nrt2.7-2* seeds. Moreover, measurement of *in situ* laccase activity excluded a possibility of the *nrt2.7-2* mutation affecting the TT10 enzymic activity at the early stage of seed development. Functional complementation of the *nrt2.7-2* mutant by overexpression of a full-length *NRT2.7* cDNA clearly demonstrated the link between the *nrt2.7* mutation and the PA phenotype. However, the PA-related phenotype of *nrt2.7-2* seeds was not strictly correlated to the nitrate content of seeds. No correlation was observed when nitrate was lowered in seeds due to limited nitrate nutrition of plants or to lower nitrate storage capacity in leaves of *clca* mutants deficient in the vacuolar anionic channel CLCa. All together, the results highlight a hitherto-unknown function of NRT2.7 in PA accumulation/oxidation.

## Summary

The seed-specific nitrate transporter AtNRT2.7 is involved in flavonoid accumulation as evidenced by the higher proanthocyanidin content in *nrt2.7-2* mutant seeds. As TT10 laccase activity is not modified, the link between NRT2.7 and proanthocyanidin accumulation has yet to be discovered.

## Introduction

Seed development and maturation lead to accumulation of N and C compounds in embryo such as reserve proteins, lipids, and carbohydrates, which are then used as energy sources during germination. The N compounds accumulated in seeds originate from nitrate (NO_3_^–^), amino acids, and peptides transferred from vegetative organs and subsequent synthesis of storage proteins. NO_3_^–^ uptake by the roots and its translocation to the aerial part and to the seeds are achieved by transporters of high-affinity and low-affinity systems (reviewed in Dechorgnat *et al.*, 2011). The high-affinity system is ensured by some members of the NRT2 family (seven members) and the low-affinity system by some members of the NRT1 family (or NPF according to the unified nomenclature proposed by Léran *et al.*, 2013; 54 members), which transport also dipeptides (reviewed in Tsay *et al.*, 2007) and other compounds such as auxin, abscisic acid, and glucosinolates (Krouk *et al.*, 2010; Kanno *et al.*, 2012; Nour-Eldin *et al.*, 2012). NO_3_^–^ uptake by roots is mediated mainly by NRT2.1 and NRT1.1 (AtNPF6.3), depending on the NO_3_^–^ concentration of the soil solution (below or above 1mM, respectively). At extremely low NO_3_^–^ concentration (below 0.025mM), NRT2.4 is also active for NO_3_^–^ uptake by roots (Kiba *et al.*, 2012). Then, root xylem loading is due to NRT1.5 (AtNPF7.3) (Lin *et al.*, 2008) and root phloem loading to NRT1.9 (AtNPF2.9) (Wang and Tsay, 2011). In shoots, xylem unloading is performed by NRT1.8 (AtNPF7.2) and NRT1.4 (AtNPF6.2) (Chiu *et al.*, 2004; Li *et al.*, 2010). In leaves, up to 50% NO_3_^–^ storage is achieved by an anion channel/transporter (chloride channel a, CLCa), which is a nitrate/proton antiporter localized in the tonoplast of foliar cells (Monachello *et al.*, 2009). Regarding the travel of NO_3_^–^ through the plant, NRT1.7 (AtNPF2.13) has been suggested as an actor in the apoplastic loading of NO_3_^–^ into the phloem sap of older leaves (Fan *et al.*, 2009). There, the delivery of NO_3_^–^ to the developing seeds is due to NRT1.6 (AtNPF2.12) located in the vascular tissue of the silique and funiculus (Almagro *et al.*, 2008). NO_3_^–^ represents quantitatively a minor N compound in dry seeds and its accumulation is due to a high-affinity NO_3_^–^ transporter, NRT2.7, specifically expressed in seeds (Chopin *et al.*, 2007). One-hour-imbibed seeds of transformants expressing a fusion between *NRT2.7* promoter and β-glucuronidase (GUS) reporter gene have shown a GUS staining in the embryo and in the endosperm. Transgenic lines carrying the GFP reporter gene fused to *NRT2.7* under the control of the 35S CaMV promoter have evidenced the tonoplastic localization of NRT2.7. NO_3_^–^ is not only an important N nutrient for plants but also a signalling molecule and the role of NO_3_^–^ in the physiology of the seed has been shown especially in breaking dormancy (Alboresi *et al.*, 2005). Mutants deficient in NRT2.7 display lower NO_3_^–^ content in dry seeds but also a higher dormancy highlighting the signalling role of NO_3_^–^ in dormancy relief (Chopin *et al.*, 2007).

Secondary metabolites such as flavonoids are synthesized during seed development and are accumulated in the seed coat and in the embryo (Lepiniec *et al.*, 2006). Flavonoids are polyphenolic compounds responsible for the brown seed colour. They have also important functions in various aspects of seed development and have health benefits when present in animal and human diet (Lepiniec *et al.*, 2006). Flavonoids are involved in protection of seeds against biotic and abiotic stresses, for instance against ultraviolet radiations, and in acting as scavengers of free radicals. The physiological functions of flavonoids in strengthening seed dormancy and viability have also been documented (Debeaujon *et al.*, 2000). Proanthocyanidin (PA) oxidation generates quinones that behave as toxic compounds against pathogens. They also constitute an antinutritive barrier against herbivores and interfere with fungal enzymes necessary for plant cell invasion. Quinones can also act as antioxidants by scavenging reactive oxygen species (ROS) produced by UV radiation, for example (reviewed in Pourcel *et al.*, 2007). *Arabidopsis* seeds contain flavonols (glycosylated aglycones derivatives) in the seed coat and embryo, and PAs or condensed tannins in the inner integument and chalaza zone (Pourcel *et al.*, 2005; Routaboul *et al.*, 2006). The biosynthesis pathway and regulations have been largely studied especially through *transparent testa* (*tt*) mutants (Lepiniec *et al.*, 2006), which are characterized by a lighter seed colour phenotype. The brown colour of *Arabidopsis* seeds occurring during desiccation is due to the oxidation of PAs and their epicatechin monomers by the laccase-like enzyme TT10/LAC15 (Pourcel *et al.*, 2005). Moreover oxidized PAs cross-link with cell-wall components, thus becoming insoluble and as such difficult to extract (Pourcel *et al.*, 2007). Seeds from the *tt10* mutant deprived of TT10 laccase-like activity are yellow at harvest but slowly darken with storage time through chemical oxidation reactions. They exhibit more soluble (i.e. extractable) PAs than wild-type seeds but are not affected in PA biosynthesis *per se*. They also accumulate less biflavonols, which are dimers of the flavonol quercetin 3-O-rhamnoside and are also synthesized by TT10. Before oxidation, PA biosynthesis and polymerization involve transport and/or vesicle trafficking (Zhao *et al.*, 2010). While the biosynthesis of PA precursors is believed to occur in the endoplasmic reticulum, transfer into the vacuole is performed by TT12 (a multidrug and toxic efflux transporter family) coupled to AHA10 a putative P-type H^+^-ATPase (Baxter *et al.*, 2005; Marinova *et al.*, 2007). However, the complete story of PA transport inside the cell has not yet been completely elucidated (reviewed in Zhao *et al.*, 2010).

This work describes a new phenotype for the *nrt2.7-2* mutant allele which exhibited seeds with more soluble PAs. Little is known about the mechanisms regulating the oxidation of tannins in seeds, and this study provides a new link between nitrogen signalling and PA metabolism. The role of NO_3_^–^ accumulated in seeds is discussed in relation to tannin oxidation, *TT10* expression, and TT10 activity.

## Materials and methods

### Plant material

The *nrt2.7-2* homozygous mutant line (EIK19) previously isolated from a T-DNA-mutagenized population of *Arabidopsis* Wassilewskija (Ws) accession in the Versailles transformant library, and the homozygous *nrt2.7.1* (*SALK_07358*) in Columbia (Col) background obtained from the ABRC stock centre (http://signal.salk.edu/cgi-bin/tdnaexpress), were both described in Chopin *et al.* (2007). The complemented lines *nrt2.7-2 C12-3* and *nrt2.7-2 C14-6* were obtained after transformation of the *nrt2.7-2* mutant by a full-length *AtNRT2.7* cDNA placed under the control of the cauliflower mosaic virus (CaMV) 35S promoter according to the method described in Chopin *et al.* (2007). The *tt10-2* mutant (CPI13 line of the Ws ecotype) was described in Pourcel *et al.* (2005) and the *tt4-8* mutant in Debeaujon *et al.* (2003). The *nrt2.7-2 tt10-2* double mutant was generated by crossing the single T-DNA-inserted mutants *nrt2.7-2* and *tt10-2*. F1 plants were grown and self-fertilized to produce a population of F2 plants and the double null mutants for NRT2.7 and TT10 were determined by PCR using primers as described in Chopin *et al.* (2007) and Pourcel *et al.* (2005). The *clca-1* and *clca-2* are T-DNA mutagenized lines isolated from the Versailles transformant library (Ws ecotype) and have been already described in Monachello *et al.* (2009).

### Growth conditions

Plants were grown in a growth chamber at 60% relative humidity with a 16/8 light/dark cycle at 21//17 °C and light intensity 150 μmol m^–2^ s^–1^. Seeds were sown on sand in 5×5cm pots and plants were subirrigated three times a week with a complete nutrient solution (10mM NO_3_^–^) containing 5mM KNO_3_, 2.5mM Ca(NO_3_)_2_, 0.25mM MgSO_4_, 0.25mM KH_2_PO_4_, 0.42mM NaCl, 0.1mM FeNa–EDTA, 30 μM H_3_BO_3_, 5 μM MnSO_4_, 1 μM ZnSO_4_, 1 μM CuSO_4_, and 0.1 μM (NH_4_)_6_Mo_7_O_24_. For the experiments on dry seeds, plants were harvested at the end of the culture, whereas for the seed development experiments, flowers at the beginning of anthesis were tagged every 3 d after fertilization (DAF) on one stalk per plant and then 6–21-d-old siliques were harvested.

For the experiment with varying nitrogen nutrition, plants were subirrigated with 10mM NO_3_^–^ from the sowing to the flowering stage and then with 0.2, 2, or 10mM NO_3_^–^. In the 2mM nutrient solution until harvest, KNO_3_ and Ca(NO_3_)_2_ concentration was 1.75mM and 0.125mM, respectively. In the 0.2mM nutrient solution, KNO_3_ concentration was 0.2mM, and Ca(NO_3_)_2_ was replaced with 0.25mM CaCl_2_.

### Nitrate content measurement

Nitrate content of seeds was determined after extraction in water of 2mg dry seeds or 1mg developing seeds excised from siliques and silique tissues (siliques without seeds). The nitrate content was measured by a spectrophotometric method adapted from Miranda *et al.* (2001). The principle of this method is a reduction of nitrate by vanadium (III) combined with detection by the acidic Griess reaction.

### C, N, total protein, amino acids, sugar, and fatty acid determination

Total C and N determination were carried out on 1mg seeds following the Dumas combustion method using a NA 1500 Serie 2 CN Fisons instrument analyser (Thermoquest) as described in Baud *et al.* (2010). Fatty acid analyses were performed on pools of 20 seeds by gas chromatography after extraction in methanol/sulphuric acid (100:2.5, v/v) as previously described (Li *et al.*, 2006). Free amino acids and sucrose contents were determined after 80% (v/v) ethanolic extraction on batches of 20 seeds according to Baud *et al.* (2002). Free amino acid content was quantified by the ninhydrin colourimetric analysis according to Rosen (1957). Sucrose was determined enzymatically using a kit (Boehringer Mannheim). Starch was quantified from the pellet resulting of the ethanolic extraction. After hydrolysis of starch by amyloglucosidase and amylase (Baud *et al.*, 2002), glucose was determined enzymatically using a kit (Boehringer Mannheim). Total protein content was determined on batches of 1.5mg seeds by the ninhydrin colourimetric quantification of the amino acids released after 1h hydrolysis of the seeds at 120 °C in 3M NaOH, as described in Baud *et al.* (2007).

### Flavonoid composition analyses

Flavonoids were extracted from 15mg dry seeds with acetonitrile/water (75:25, v/v), as described in Routaboul *et al.* (2006). After centrifugation of the extracts, the supernatant was used for the analysis of flavonols and soluble PAs, while the pellet contained insoluble PAs. Analyses of soluble and insoluble PAs were further performed after acid-catalysed hydrolysis and absorbance measured at 550nm using cyanidin as a standard molecule according to Routaboul *et al.* (2006). Epicatechin monomers and PA polymers were then analysed by LC-MS. Flavonol composition was also analysed by LC-MS using apigenin as an internal standard which was added at the time of extraction (Routaboul *et al.*, 2006).

### RNA extraction and gene expression analysis

Total RNA was extracted from excised seeds or siliques having their seeds removed (the material from three siliques at the same development stage were pooled for each extract) with a RNeasy Plant Mini kit (Qiagen). First-strand cDNAs were synthesized from 1 μg RNA using Moloney murine leukaemia virus reverse transcriptase (Thermo Scientific) and oligo(dT)15 primers (Thermo Scientific). The absence of DNA contamination was verified by PCR using specific primers spanning an intron in *Nii* (At2g15620): forward 5′-TGCTGATGACGTTCTTCCACTCTGC-3′; reverse 5′-CTG AGG GTT GACTCCGAAATA GTCTC-3′. Gene expression analyses were determined by quantitative real-time PCR (qPCR, Eppendorf Realplex MasterCycler with a Roche LightCycler-FastStart DNA Master SYBR Green I kit, according to the manufacturer’s protocol) using 2.5 μl of a 1:5 dilution of first-strand cDNA in a total volume of 10 μl. The gene-specific primers were: TT10 (At5g48100): forward 5′-GCCAGAGCTTA CCAAAGCGG-3′, reverse 5′-CCAAAAGCT GCTGAGGTGTCAT-3′; NRT2.7 (At5g14570): forward 5′-CCTT CATCCTCGTCCGTTTC-3′; reverse 5′-AATTCGG CTATGGTGG AGTA-3′; CLCa (At5g40890) forward 5′-TCACACATCGAGA GTTTAGATT-3′; reverse 5′-AATGTAGTAGCCGACGGCGAG AA-3′. The results were standardized using two reference genes chosen as the most stable and accurate ones during seed maturation: *EF1α* (At5g60390: forward 5′-CTGGAGGTTTTGAGG CGGTA-3′; reverse 5′-CAAAGGGTGAAAGCAAGAAGA-3′) and *APC2* (At2g04660: forward 5′-GAAACATCAATTGCCTCTGTG GAAGA-3′ and reverse 5′-AAGGATCAGCCACA CAAAACATC TTG-3′). The expression of each gene was normalized to the level of a synthetic reference gene (SRG) as follows (Vandesompele *et al.*,) 2002


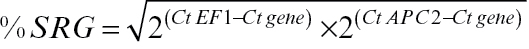


### In situ TT10 activity

The *in situ* enzymic activity of TT10 was measured as described in Pourcel *et al.* (2005). The accelerated browning assay was performed on immature seeds (7–8 DAF) in 100mM phosphate buffer pH 6.6, 50mM epicatechin (Sigma-Aldrich). Vacuum was applied for 1h before incubation at 37 °C in the dark overnight. Seeds were observed directly under a binocular for relative browning intensities.

## Results and discussion

### Soluble PAs are more accumulated in seeds of the *nrt2.7-2* mutant

**Fig. 1.**
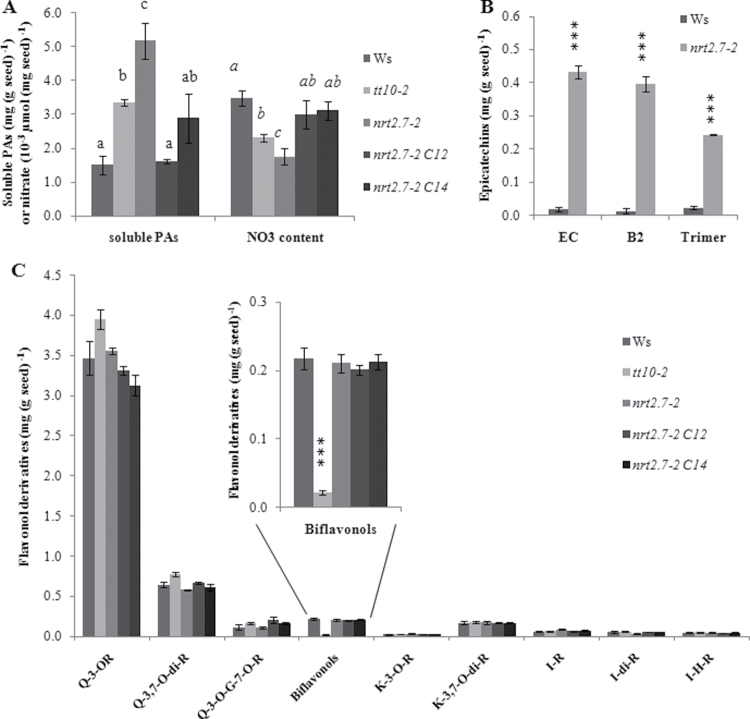
Nitrate content and flavonoid composition of *tt10-2*, *nrt2.7-2*, nrt2.7-*2 C12*, *nrt2.7-2 C14*, and wild-type (Ws) mature seeds. (A) Analysis of soluble proantocyanidins (PAs) after acid-catalysed hydrolysis and determination of nitrate content. (B) Analysis of epicatechin (EC) monomers and oligomers (B2 and trimer) by LC-MS. (C) Analysis of flavonol composition by LC-MS. G, Glucoside; H, hexoside; I, isorhamnetin; K, kaempferol; Q, quercetin; R, rhamnoside. Values are mean±standard error of seeds of three individual plants. Statistical analysis was performed using analysis of variance and the means were classified using Tukey HSD test (*P*<0.05): (A) a, b, c, different letters above bars indicate statistically significant differences; (B, C) *** indicates significant differences between Ws and the *nrt2.7-2* mutant or between Ws and the *tt10.2* mutant (Student t-test *P*<0.001).

Because NO_3_^–^ is an important N nutrient for plants, the impact of the lack of NRT2.7 on the accumulation of other N and C reserve compounds was evaluated in *nrt2.7* mutant seeds when grown on nonlimited supply of N (10mM NO_3_^–^). Total N and free amino acid contents were not affected in the *nrt2.7-2* mutant compared to the wild type (Table 1), while total protein content was slightly increased (Table 1) and NO_3_^–^ content was decreased (Fig. 1A). A decrease in NO_3_^–^ content was also observed in *tt10-2* mutant seeds (Fig. 1A). In contrast, total C, fatty acids, starch, and sugar contents were not changed in the *nrt2.7-2* mutant (Table 1). Thus, the decrease in capacity to store NO_3_^–^ in *nrt2.7-2* seed vacuole seemed to favour the accumulation of N-protein reserve compounds without affecting C content.

**Table 1.** Analysis of mature seeds from Ws and the *nrt2.7-2* mutantTotal N and C contents, total protein, soluble amino acids, sugars, starch, and total fatty acids were determined on seeds of plants grown on 10mM NO_3_^–^. Values are mean±SE of seeds of three or four individual plants. * indicates significant differences between the wild type (Wassilewskija, Ws) and the *nrt2.7-2* mutant (Student t-test *P*<0.05).

Parameter	Ws	*nrt2.7-2*
N (% seed dry weight)	3.75±0.04	3.78±0.01
Total protein (μg BSA (mg seed)^–1^)	140.53±6.63	181.98±13.04*
Soluble amino acids (nmol (mg seed)^–1^)	10.68±3.25	11.01±4.41
C (% seed dry weight)	54.04±0.31	53.19±0.24
Fatty acids (μg (mg seed)^–1^)	378.27±1.46	371.44±4.15
Sucrose (nmol (mg seed)^–1^)	35.88±1.94	34.59±0.40
Starch (eq nmol Glu (mg seed)^–1^)	2.60±0.17	2.48±0.22

**Fig. 2.**
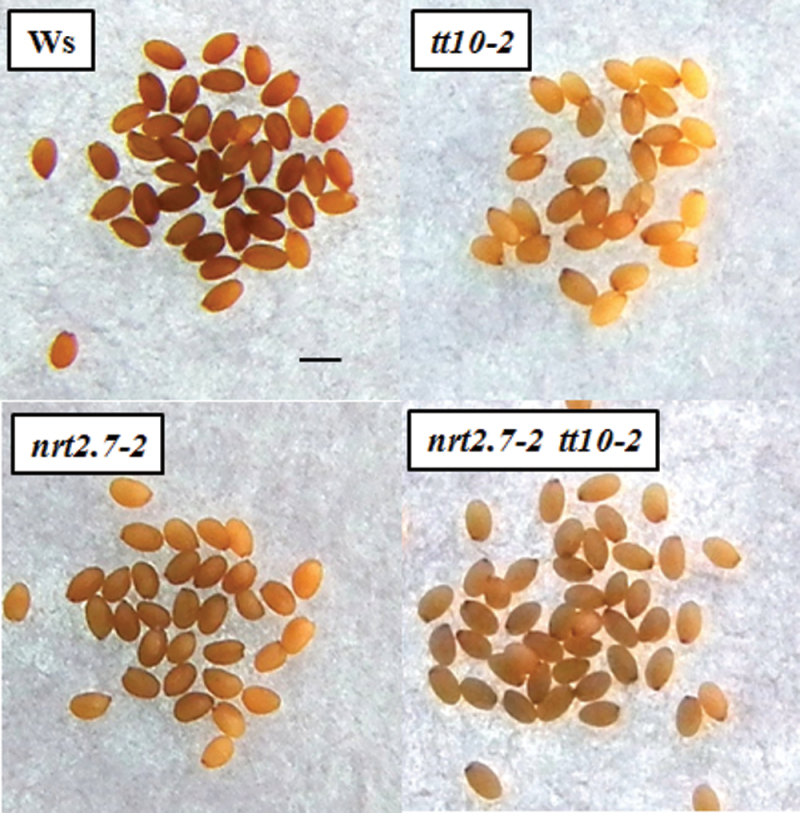
Colour phenotype of mature seeds of *nrt2.7-2* and *tt10-2* simple mutants, *nrt2.7-2 tt10-2* double mutant, and wild-type (Ws) mature seeds. Mother plants were grown on 10mM NO_3_^–^ and seeds were observed at harvest. Bar, 500 μm.

Interestingly the *nrt2.7-2* mutant seeds displayed a slightly lighter colour compared to the wild-type Ws, resembling the phenotype of *tt10* mutant seeds (Pourcel *et al.*, 2005) (Fig. 2). This lighter colour phenotype was also observed in the double mutant *nrt2.7-2 tt10-2* with a pale-brown seed coat colour and a dark-brown chalaza zone (Fig. 2). The analysis of flavonoids in mature seeds revealed that the soluble PA content was similarly increased in the *nrt2.7-2* and *tt10-2* mutants compared to Ws seeds (Fig. 1A) while insoluble PAs were not changed (Supplementary Fig. 1, available at *JXB* online). LC-MS analyses showed that the soluble epicatechin monomers and oligomers were also increased in the *nrt2.7-2* mutant (Fig. 1B) as well as in the *tt10-2* mutant (see Pourcel *et al.*, 2005). However, unlike the *tt10-2* mutant that contains only very small amounts of biflavonols (dimer of quercetin 3-O-rhamnoside) and a slightly more quercetin 3-O-rhamnoside monomers (Fig. 1C), the flavonol composition of the *nrt2.7-2* mutant was not modified (Fig. 1C). Thus, the *nrt2.7-2* mutant was very peculiar as it exhibited a modification in flavonoid composition that was specific for PAs and perhaps, as in the *tt10* mutant, linked to a defect in PA oxidation. It remains to be investigated whether the TT10 function is altered in *nrt2.7-2* or whether another oxidative mechanism is involved.

To first confirm the link between the T-DNA insertion in *At5g14570* and the phenotype of *nrt2.7-2*, a functional complementation of *nrt2.7-2* was conducted with a construct, *Pro35S:AtNRT2.7*, which allows overexpression of a full-length *NRT2.7* under control of the CaMV 35S promoter. As a result, the soluble PAs and nitrate content phenotypes were both restored in the two complemented lines *nrt2.7-2 C12* and *nrt2.7-2 C14* (Fig. 1A). However, the PA phenotype of the *nrt2.7-2* mutant allele was specific for the Ws accession, since no difference in soluble PAs was observed in the *nrt2.7-1* null mutant allele in Columbia background (Supplementary Fig. S2 available at *JXB* online) whereas the nitrate phenotype was also encountered in *nrt2.7-1* (Chopin *et al.*, 2007). These data suggest that nitrate and soluble PA contents are not directly correlated. The specificity of the PA phenotype for the Ws accession was surprising but natural variability in PA accumulation has already been reported (Lepiniec *et al.*, 2006; Routaboul *et al.*, 2012), suggesting a variability in the regulation of PA oxidation. Besides, plant nitrate content varies also among accessions and, more precisely, Col accession displays a higher capacity to store nitrate than Ws accession in seeds (Chopin *et al.*, 2007) and in foliar tissues (North *et al.*, 2009), and consequently Col is more tolerant to N limitation. Control of PA oxidation originating from natural diversity of strategies for nitrate use and storage might explain the lack of PA phenotype for the Col accession.

**Fig. 3.**
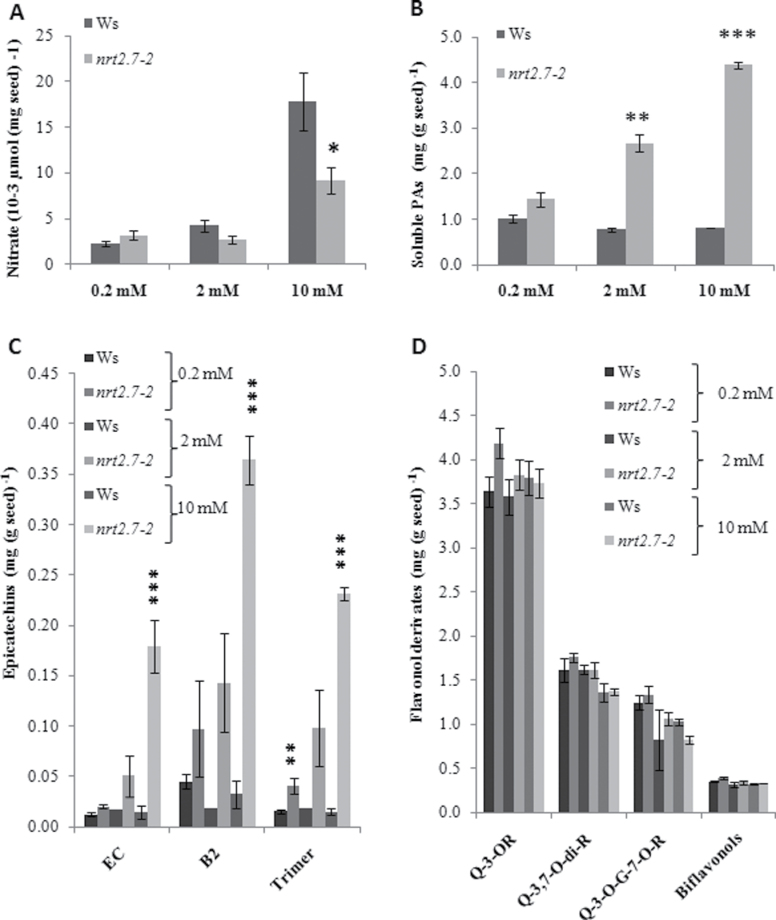
Nitrate content and flavonoid composition of *nrt2.7-2* and wild-type (Ws) mature seeds under various nitrate nutrition levels. (A) Nitrate content. (B) Soluble proantocyanidins (PAs) after acid-catalysed hydrolysis. (C) Epicatechin (EC) monomers and oligomers (B2 and trimer) by LC-MS. (D) Flavonol composition by LC-MS. G, Glucoside; Q, quercetin; R, rhamnoside. Values are mean±standard error of seeds of three individual plants. Significant differences between Ws and the *nrt2.7-2* mutant (Student t-test): *P<0.05, **P<0.01, ***P<0.001.

Thus, this work investigated further the relationship between nitrate accumulated in the seed and condensed PA accumulation. Since the PA phenotype of the *nrt2.7-2* mutant was first observed when plants were grown on nonlimiting supply of N nutrition (10mM) as described above, a more comprehensive range of NO_3_^–^ nutrition was also tested from 0.2 and 2mM NO_3_^–^ as limited N levels to 10mM NO_3_^–^. The NO_3_^–^ content of dry seeds was linked to the NO_3_^–^ nutrition in both genotypes and it was lower in the *nrt2.7-2* mutant than in Ws on 10mM NO_3_^–^, but not significantly affected on 2 and 0.2mM NO_3_^–^ (Fig. 3A). Epicatechin and soluble PAs (epicatechin oligomers) were more accumulated in the *nrt2.7-2* mutant for all nutrition levels (Fig. 3B and C), while still no change was observed for flavonols (Fig. 3D). The effect of *nrt2.7-2* mutation on both NO_3_^–^ and soluble PA contents increased with the NO_3_^–^ nutrition level. Considering these results, subsequent experiments were performed at 10mM NO_3_^–^, which allowed viewing of the most pronounced flavonoid and nitrate phenotypes.

**Fig. 4.**
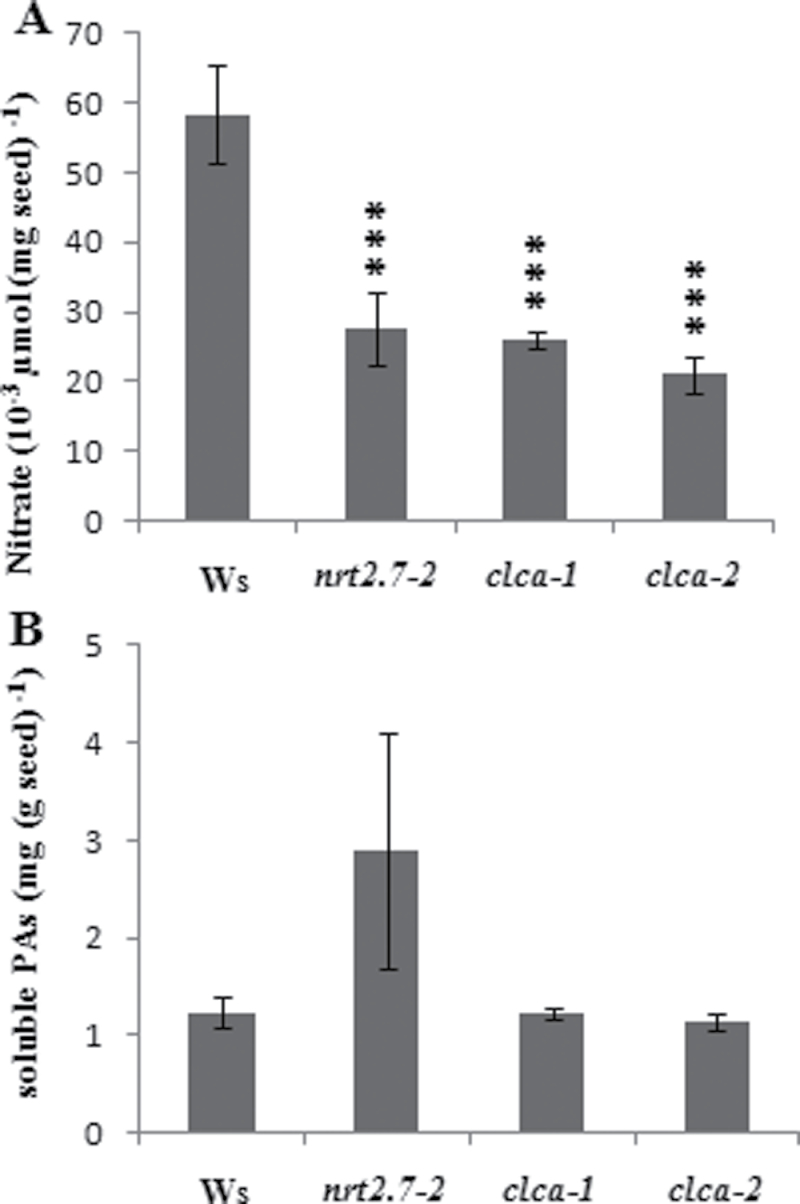
Nitrate and soluble proantocyanidin (PA) contents of *nrt2.7-2*, *clca-1*, *clca-2*, and wild-type (Ws) mature seeds. (A) Nitrate content. (B) Soluble PAs after acid-catalysed hydrolysis. Values are mean±standard error of seeds of three individual plants. Significant differences between Ws and the *nrt2.7-2* mutant (Student t-test): ***P<0.001.

The NO_3_^–^ content in seeds was dependent on supply of NO_3_^–^ nutrition (Fig. 3A) and thus may be relevant to the NO_3_^–^ availability for allocation to the seeds. This work speculated whether a limited capacity of NO_3_^–^ storage in leaves could also modulate NO_3_^–^ transfer to the seeds and could also influence the soluble PA level in seeds. Therefore, this work analysed the consequence of a knockout mutation in *CLCa*, encoding a nitrate/proton antiporter responsible for NO_3_^–^ accumulation in vacuolar compartment in leaves (Monachello *et al.*, 2009). Interestingly, NO_3_^–^ content was decreased in *clca1* and *clca2* mutant seeds to the same extent as in the *nrt2.7-2* mutants, while the soluble PA accumulation was not changed in *clca1* and *clca2* mutants (Ws background) (Fig. 4A and B). This result suggested that the mechanism linking NO_3_^–^ accumulation and PA accumulation in seeds was specifically linked to *NRT2.7* function in seeds rather than to global NO_3_^–^ accumulation.

### Nitrate accumulation during seed development

**Fig. 5.**
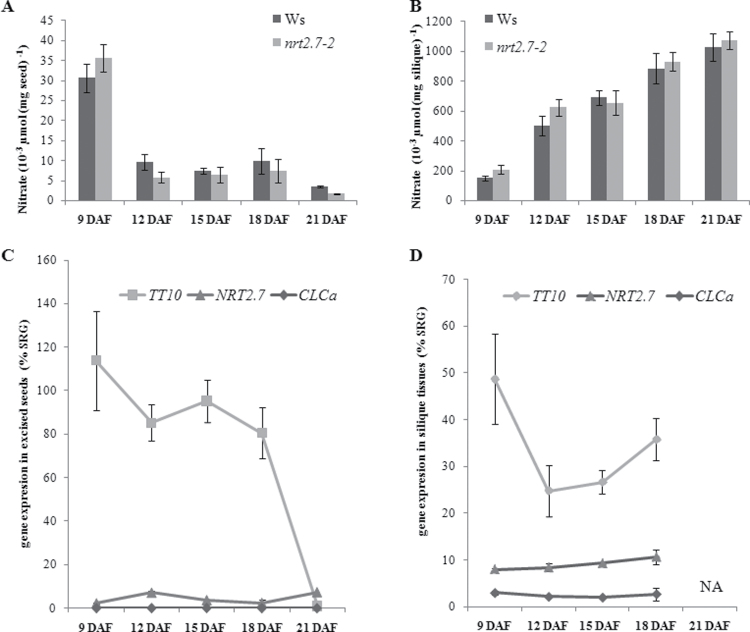
Nitrate content and gene expression in developing seeds from 9 to 21 d after flowering (DAF). (A, B) Nitrate content of *nrt2.7-2* and wild type in excised seeds (Ws) (A) and in siliques emptied from their seeds (silique tissues) (B). (C, D) Expression of *TT10*, *NRT2.7*, and *CLCa* of Ws in excised seeds (A) and silique tissues (B). Each gene expression data was normalized to the level of a synthetic reference gene (SRG) using reference genes *EF1a* and *APC*, as described in Materials and methods. Values are mean±standard error of seeds of three individual plants. NA, not analysed.

It has already been described that PA oxidation in the testa starts with the desiccation of developing seeds (Pourcel *et al.*, 2005). In order to better understand the link between NRT2.7 and PA oxidation/accumulation in seeds, the current work investigated more precisely the fluctuation of NO_3_^–^ content in seeds and in siliques tissues (siliques excluding seeds) during seed development. The NO_3_^–^ content was the highest in young seeds (9 DAF) and decreased abruptly (12 DAF) to the final low content in mature seeds (Fig. 5A). Conversely NO_3_^–^ content was the lowest in young siliques tissues (9 DAF) and increased regularly up to the senescing stage (21 DAF) (Fig. 5B). In the *nrt2.7-2* mutant, the NO_3_^–^ contents were slightly lowered in seeds at 12 DAF and in mature seeds compared to those in Ws (Fig. 5A), concomitantly to the maxima of *NRT2.7* expression in Ws (Fig. 5C). In contrast, NO_3_^–^ content was not affected in silique tissues of the *nrt2.7-2* mutant (Fig. 5B). Thus, NRT2.7 was likely not the only actor responsible for NO_3_^–^ accumulation in these tissues. According to Almagro *et al.* (2008), the impact of the *NRT1.6* (*AtNPF2.12*) mutation was strongly associated with a reduced NO_3_^–^ content in seeds and an increased seed abortion, but no colour phenotype of the *nrt1.6* mutant seeds was reported. In the current study, no significant difference in *NRT1.6* (*AtNPF2.12*) expression was measured in Ws and in *nrt2.7-2* (data not shown). *NRT1.6* (*AtNPF2.12*) was expressed in the vascular tissue of the silique and funiculus and was partially responsible for the delivery of NO_3_^–^ into the seed, but *NRT1.6* (*AtNPF2.12*) was localized at the plasma membrane and, thus, may not be able to compensate the vacuolar nitrate storage in *nrt2.7-2.* Expression of the vacuolar anionic channel CLCa was detected in silique tissues (Fig. 5D) and, thus, could explain the partial compensation mechanism for the loss of NRT2.7 function in this organ, but no expression of CLCa was measured in excised seeds (Fig. 5C). Further study is required to find out if any other transporter is functional in these organs.

### The PA phenotype of the *nrt2.7-2* mutant is not due to a modulation of TT10 expression

*nrt2.7-2* mutant seeds accumulated less NO_3_^–^ and more soluble PAs and epicatechins compared to Ws partially resembling *tt10* mutant phenotype. Thus, this phenotype was likely arising from a defect in PA oxidation leading to an accumulation of soluble forms of PAs during the development. According to Pourcel *et al.* (2005), *TT10* expression in entire siliques begins to be detected at 4 DAF. Thus, the current work investigated *TT10* and *AtNRT2.7* expression in excised seeds and silique tissues excluding seeds of Ws and the *nrt2.7-2* mutant during seed development. In Ws, the level of *NRT2.7* mRNA was lower than *TT10* but they were expressed in seeds and siliques (Fig. 5C and 5D). The expression patterns of *TT10* and *NRT2.7* varied along seed development. *TT10* expression was repressed in excised seeds from 9 DAF to 21 DAF (or mature seeds) (Fig. 5C). *TT10* mRNA levels in silique tissues were measured 50% lower than those in excised seeds (Fig. 5D). In contrast, *NRT2.7* expression showed two maxima in excised seeds, at 12 and 21 DAF (Fig. 5C) and increased slightly in silique tissues from 9 to 18 DAF (Fig. 5D). Furthermore, this work failed to observe a modified expression pattern of *TT10* that was significantly reproducible in the *nrt2.7-2* mutant compared to Ws (data not shown).

A role in signalling was previously suggested for NO_3_^–^ in relieving seed dormancy (Alboresi *et al.*, 2005). However, considering that the maximum of *TT10* expression preceded the first raise in *NRT2.7* expression and the beginning of NO_3_^–^ content to decrease in *nrt2.7-2*, the current study excluded the hypothesis of a signalling role for NO_3_^–^ in downregulating the expression of *TT10* and then lowering soluble PA oxidation.

### Is the PA phenotype of the *nrt2.7-2* mutant due to a modulation of TT10 activity?

In order to find out a causal explanation for the PA phenotype of the *nrt2.7-2* mutant, TT10 activity was considered. The enzymic activity of TT10 has never been successfully measured *in vitro* but an assay for *in situ* detection of browning in immature seed coat has been reported by Pourcel *et al.* (2005). In a first attempt, the current study looked into the *in situ* measurement of TT10 activity in young seeds (7–8 DAF) of Ws and the *nrt2.7-2* mutant using the *tt10-2* mutant as a negative control and the *tt4-8* mutant as a positive control without endogenous supply of flavonoids (due to the lack of chalcone synthase). The browning intensity of the seeds incubated in presence of the epicatechin substrate revealed the PA oxidation activity of TT10. As expected, *tt10-2* seeds stayed colourless and seeds of Ws and *tt4-8* showed a brown colour, but *nrt2.7-2* seeds became as brown as Ws (Table 2). These results suggested that the oxidative activity of TT10 was not altered in *nrt2.7-2* seeds at this stage. However, this type of experiment is only feasible when the testa was still colourless in immature seeds. At this stage *TT10* was highly expressed but these conditions were not favourable for a maximal *NRT2-7* expression. Further investigation of TT10 activity by optimizing the *in situ* measurement at older stages is needed to understand the mechanism of higher soluble PA accumulation in *nrt2.7-2* seeds.

**Table 2.** *In situ* enzymic activity of the TT10 laccase in the wild type and mutantsThe analysis was performed according to the method described in Pourcel *et al.* (2005). The table describes seed coat colour with and without (control) the addition of epicatechin substrate to immature seeds (7–8 DAF). The browning colour intensity is positively correlated to TT10 activity. It is recorded by visual observation and noted as such: –, colourless; +++<++++, increasing browning colour. Approximately 50 seeds per sample were analysed.

Substrate	Ws	nrt2.7-2	tt10.2	tt4.8
Control	–	–	–	–
Epicatechin	+++	+++	–	++++

Since the mechanisms for regulating the TT10 activity are largely unknown, the link between NRT2.7 and TT10 activity is difficult to assess. TT10 protein has been described as a putative laccase containing four His-rich copper-binding domains, corresponding to the putative catalytic sites of the multi-copper oxidase family (Pourcel *et al.*, 2005). A phylogenetic analysis has revealed the highest homology of TT10 with four other dicotyledonous laccases (and for example with RvLAC2 from the sap of the Japanese lacquer tree *Rhus vernicifera*). Nitric oxide (NO) has been reported as a regulator of laccases, acting as a reducer of the *R. vernicifera* laccase RvLAC2 and also of fungal laccases (Torres and Wilson, 1999; Wilson and Torres, 2004). However, the consequences of the NO action on the enzymic activity of laccase are not completely understood (Torres *et al.*, 2002). TT10 protein has recently been experimentally shown to be localized in vacuole (Pang *et al.*, 2013), the same cellular compartment as NRT2.7, but a hypothetical link between NO, TT10 activity, and NRT2.7 remains uncertain.

### What is a role for NRT2.7 in PA oxidation/accumulation?

According to Pourcel *et al.* (2005), *TT10* is expressed in the developing testa, firstly in the inner integument (PA-producing cells) and afterwards in the outer integument (location of flavonol synthesis). *NRT2.7* expression has been previously localized in the endosperm and in embryo in imbibed seeds (Chopin *et al.*, 2007) while PAs are synthesized and accumulated in the endothelium. Although the current work was able to measure *NRT2.7* expression by qPCR in excised seeds, all attempts viewing the localization of NRT2.7 in the seed during its development by *in situ* hybridization or immunolocalization were unsuccessful. However, *NRT2.7* expression is present in the seed coat according to the data available on the eFP browser web site (http://bbc.botany.utoronto.ca/efp_seedcoat/cgi-bin/efpWeb.cgi). NRT2.7 has already been described as a NO_3_^–^ transporter (Chopin *et al.*, 2007), which is coherent with the lower NO_3_^–^ content in seeds of *nrt2.7* mutant. Since the PA phenotype appeared more strictly correlated to the presence of NRT2.7 than to the vacuolar NO_3_^–^ content (Fig 3A and B), it was speculated whether the function of NRT2.7 in PA oxidation could be related to another function of NRT2.7 hitherto unknown. It has been demonstrated that NRT1 (NPF) proteins are able to transport molecules other than nitrate (Léran *et al.*, 2013), although little is known about the NRT2 family. Further experiments are needed in order to ascertain such hypothesis. The transport of epicatechin into and out of the vacuolar compartment could have been disturbed in absence of NRT2.7. TT12 is a MATE transporter involved in the storage of PA precursor into the vacuole and its activity is coupled to AHA10, an H^+^-ATPase. *Aha10* and *tt12* mutants are affected in PA accumulation and also in the vacuolar biogenesis, supporting an endomembrane function for these transporters. There may be a direct or indirect link between these transport activities and NRT2.7 that involves pH stability, tonoplast stabilization, or other unknown mechanism.

## Supplementary Material

Supplementary Data
