# Supplementary material for: Proanthocyanidin oxidation of Arabidopsis seeds is altered in mutant of the high-affinity nitrate transporter NRT2.7
Source: J Exp Bot. 2014 Feb 13;65(3):885–93. doi: 10.1093/jxb/ert481 (PMC3924729; doi:10.1093/jxb/ert481)
Supplement: Supplementary Data [file supp_65_3_885__index.html]

Proanthocyanidin oxidation of Arabidopsis seeds is altered in mutant of the high-affinity nitrate transporter NRT2.7 — Supplementary Data 

# Proanthocyanidin oxidation of *Arabidopsis* seeds is altered in mutant of the high-affinity nitrate transporter NRT2.7

## Supplementary Data

Data files

**Files in this Data Supplement:**

- Supplementary Data - Supplementary Data
