# Supplementary material for: Proanthocyanidin oxidation of Arabidopsis seeds is altered in mutant of the high-affinity nitrate transporter NRT2.7
Source: J Exp Bot. 2014 Feb 13;65(3):885–93. doi: 10.1093/jxb/ert481 (PMC3924729; doi:10.1093/jxb/ert481)

Title

Proanthocyanidin oxidation of Arabidopsis seeds is altered in mutant of the high affinity nitrate transporter NRT2.7.

Authors

Laure C. David[1*] and Julie Dechorgnat[2*], Patrick Berquin[1], Jean Marc Routaboul[3], Isabelle Debeaujon[1], Françoise Daniel-Vedele[1], Sylvie Ferrario-Méry[1,]

[1] Institut Jean-Pierre Bourgin (IJPB), UMR 1318 INRA-AgroParisTech, Centre de Versailles-Grignon, Route de St-Cyr (RD10), F-78026 VERSAILLES cedex, France

[2] University of Adelaide, School of Agriculture Food and Wine, PRC, 2B Hartley Grove, Urrbrae,SA 5064 Australia

[3] Genomic and Biotechnology of Fruit, UMR 990 INRA/INP-ENSAT, 24, Chemin de Borderouge-Auzeville CS 52627, F-31326 CASTANET-TOLOSAN Cedex, France

* These authors contributed equally to this study

Corresponding author:

Sylvie.Ferrario@versailles.inra.fr

tel: +33(1) 30 83 30 94

fax: +33(1) 30 83 30 96

supplementary Figure 1: Analysis of insoluble PAs of *nrt2.7-2* and wild type (Ws) mature seeds after acid-catalyzed hydrolysis. Values are the means ± SE of seeds of three individual plants.

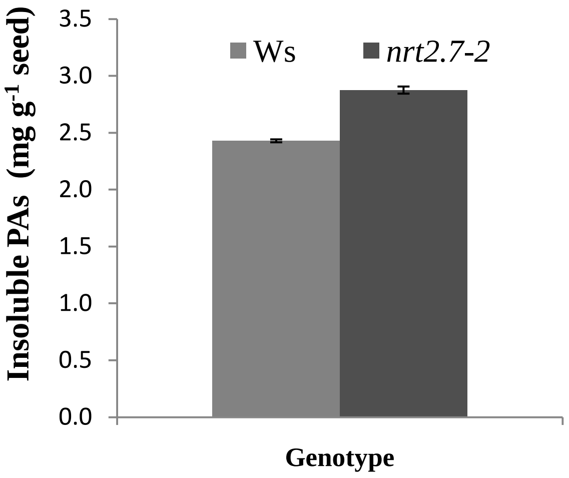

supplementary Figure 2: Analysis of soluble PAs of *nrt2.7-1* and wild type mature seeds (Col accession). Values are the means ± SE of seeds of three individual plants.

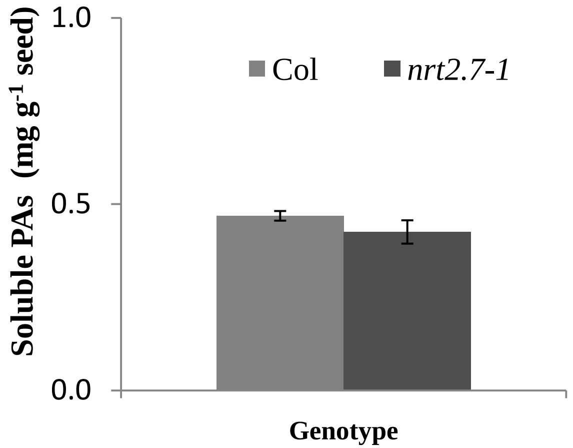

Supplement: Supplementary Data [file supp_ert481_jexbot112953_file001.pdf]
